# Supplementary material for: A molecular neuromorphic network device consisting of single-walled carbon nanotubes complexed with polyoxometalate
Source: Nat Commun. 2018 Jul 12;9:2693. doi: 10.1038/s41467-018-04886-2 (PMC6043547; doi:10.1038/s41467-018-04886-2)
Supplement: Supplementary file 1 — Supplementary Information [file 41467_2018_4886_MOESM1_ESM.pdf]

Supplementary Information for

A Molecular Neuromorphic Network Device consisting of Single-Walled Carbon Nanotubes complexed with Polyoxometalate

Hirofumi Tanaka, *et al*.

Contents

Supplementary Notes 1: Impulse generation in a POM/SWNT complex device with a microscale channel

Supplementary Notes 2: Rudimentary reservoir computing with SWNT/POM network device model

Supplementary Notes 3: Possible experimental considerations for a reservoir computing system consisting of a POM/SWNT complex

Supplementary Note 1: Impulse generation in a POM/SWNT complex device with a microscale channel

We recently confirmed that a microscale POM/SWNT complex device generated current impulses. As shown in Supplementary Figs. 1a and 1b, SWNTs served as bridges between Au electrodes with a 1-μm gap length, where the SWNTs were aligned by using the electrophoresis method. POMs were dosed into the device after fabrication of the SWNT device. The fabrication details are described in a reference paper[S1] including a study of stochastic-resonance device fabrication by using spontaneous noise generation in a POM/SWNT complex system.

Supplementary Figure 1c shows time domain current for 0.5 s with a DC bias voltage of 0.5 V across the electrodes. In our previous study for noise generation, we found that even a device whose POM density was higher than that appropriate for noise generation, generated current impulses.

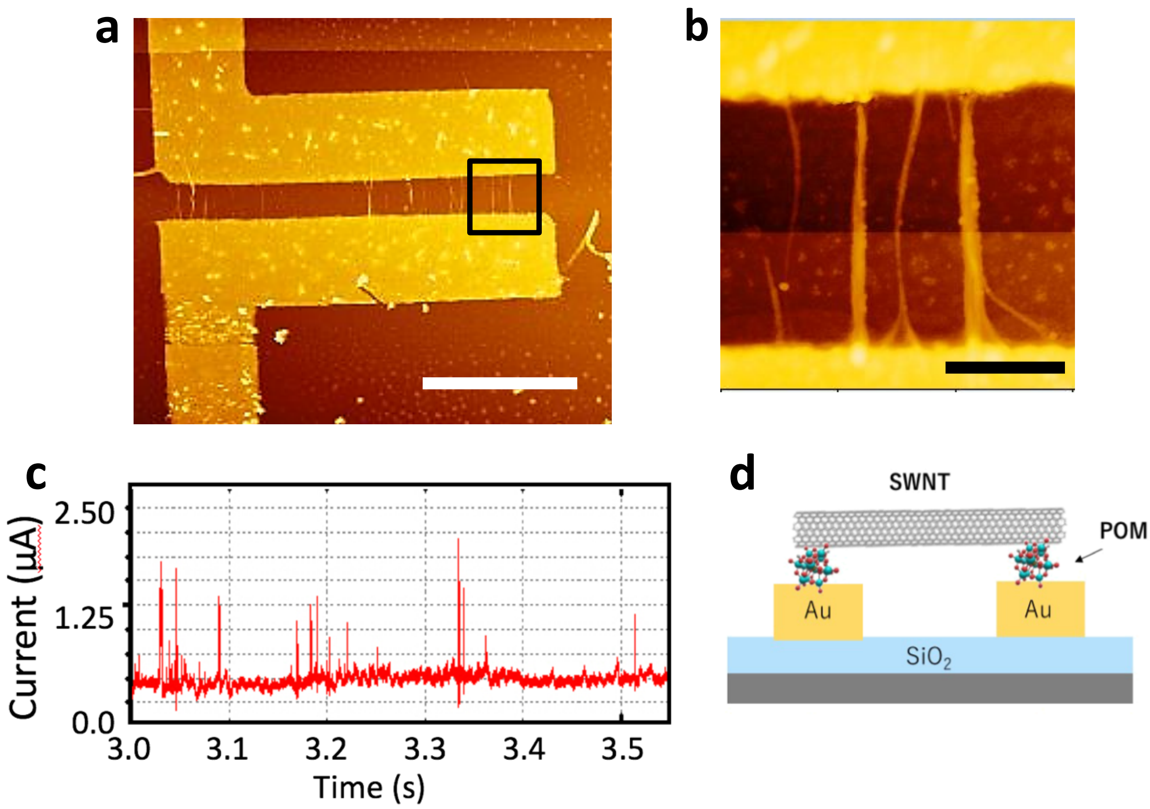

Supplementary Figure 1 | (**a**) AFM image of the device (scale bar = 5 μm). (**b**) Enlarged AFM image of the SWNTs between the electrodes inside the black square in (**a**) (scale bar = 0.5 μm). (**c**) Time-domain current of the device biased by applying 0.5 V across its electrode. (**d**) Schematic cross-sectional view of the device.

We consider that the current impulses to originate from the POMs inserted between the SWNTs and Au electrodes shown in Supplementary Fig. 1d. Based on the previous study, we know that POMs adsorbed onto the SWNTs generate noise rather than impulses. The conductance change of the observed impulses was three orders of magnitude greater than that of the noise. Hence, conductance switching at the POM junction should be the origin of the impulses in these small size devices.

The current impulses generated by the small-sized POM/SWNT device (having the electrodes with 1-μm gap length shown in Supplementary Fig. 1) were analogous to those generated by a large-sized POM/SWNT device (having the electrodes with 1-mm gap length shown in Fig. 2b), including the spike frequencies and amplitudes (current scale of microamperes). The electric field has almost the same order of magnitude in both devices: $150\ \mathrm{V}/\ 1\mathrm{mm} = 1.5 \times 10^5$ (V/m) for the large-sized device (generating impulses with 150 V bias voltage, as in Fig. 3b), and $0.5\ \mathrm{V}/1\mu\mathrm{m} = 5 \times 10^5$ (V/m) for the small-sized device (also generating impulses with 0.5 V bias, as in Supplementary Fig. 1c).

Supplementary Note 2: Rudimentary reservoir computing with SWNT/POM network device model

We here demonstrate rudimentary *reservoir computing* on the POM/SWNT device model. Supplementary Figure 2a depicts the typical structure of a reservoir system with external feedback. A recurrent generator network (blue spheres and directed arrows in Supplementary Fig. 2a) with firing rates $\mathbf{r}$ generates complex nonlinear dynamics, and drives a linear readout unit Σ with output z through weights $\mathbf{w}$ (red) that are modified during learning. The output z is fed back to the reservoir, to maintain the complex nonlinear dynamics in the reservoir. Note that only the connections shown in red are subject to modification during learning.

Our 2D POM/SWNT network model is used as a reservoir, as shown in Supplementary Fig. 2b. The external feedback is implicitly introduced into this reservoir system, where its feedback is created by connecting the external bias voltage source V_{B} across the electrodes, which results in maintaining and generating noisy or spiking dynamics, as exhibited in Fig. 4f. Among all of the POM particles (small blue spheres in Supplementary Fig. 2b), R particles are randomly selected, and the charges are read out by virtual probes (red lines in Supplementary Fig. 2b). The charges read out at time t, $\left(r_1(t),\cdots,r_R(t)\right)$ ($\equiv \mathbf{r}(t)$) drive the readout unit Σ through weights $\left(w_1(t),\cdots,w_R(t)\right)$ ($\equiv \mathbf{w}(t)$), and the reservoir output is given by $z(t) = \mathbf{w}^{\mathrm{T}}(t)\mathbf{r}(t)$. During learning, a temporal signal $s(t)$ is applied to the system as a supervisor. By using the FORCE learning algorithm[S2], the weight is updated to attain $z(t) \approx s(t)$.

Extensive numerical simulations were conducted to reveal the fundamental properties of our reservoir system. Here, the NARMA10 sequence[S3], one of the most widely used benchmarks in reservoir computing, was used as the supervisor. Supplementary Figure 2c presents one of the results when $R = 100$ with 5,500 POM particles and 4,500 defects on a 100×100 rectangular grid ($D_f = 45\%$), where the readout output $z(t)$ (purple) is plotted, while the supervisor (NARMA10 sequence; green) is superimposed, exhibiting that the reservoir system could memorize a part of the NARMA10 temporal sequences essentially through the FORCE learning, by utilizing complex dynamics generated by the POM/SWNT network.

The quality of memory function is determined by the replicability of the given data as well as the data length. To reveal the quality, we evaluated the normalized root-mean-square deviation (NRMSD) between the supervisor $s(t)$ and generated output $z(t)$ after learning. Supplementary Figure 2d shows NRMSD versus the signal length for two different R values (50 and 100). The NRMSD increases gradually as the

signal length increases, whereas larger R decreases the NRMSD, indicating that the quality of memory function is improved by increasing R.

In the simulations described above, R particles were randomly selected from all of the particles in the device. Supplementary Figure 2e shows yet another method of the random particle selection, where particles are randomly selected from areas beside the source electrode (upper, labelled by ‘source side’) or the drain electrode (bottom, ‘drain side’). Such topological deflection upon random particle selection may influence the performance of the reservoir system due to the network complexity difference, because the dynamics of the particles sampled near the source electrode are generated by local interactions in the source-side (shallow) network, whereas those of the particles sampled nearby the drain electrode are generated by deep interactions among particles from the source to the drain side. Supplementary Figure 2f compares the NRMSDs in the cases of source- and drain-side particle sampling as functions of the normalized sampling area ($\equiv l$) when $R = 100$. Both of the NRMSDs monotonically decreased as l increases. A significant difference between the NRMSDs is observed when $l < 0.2$, whereas the values are almost the same when $l > 0.2$, which indicates that the source-side sampling is much better than the drain-side sampling when the placement of readout wires is limited around the source or drain electrode ($l < 0.2$); however, to minimize the NRMSD, the particles must be sampled from particles located anywhere in the device ($l = 1$).

Our POM/SWNT network device imitates the spiking behaviours of complex neural networks; however, does not include any synaptic function. One of the difficult challenges is to include synaptic functions in POM/SWNT networks for effective neuromorphic demonstrations. One possible method is to complex memristive molecules, such as BPDN molecules[S[4]], in the POM/SWNT network so that POMs (as spiking neurons), memristive molecules (as synapses), or both, can be localized at the SWNT junctions; however, it might take a very long time to find computational and useful functions. On the other hand, in our reservoir framework, the reservoir itself (POM/SWNT networks as complex spiking networks) and the external synaptic readout wires are separated; hence, one can get one of the useful reservoir functions, *i.e.* temporal coding of complex time series, by controlling the synaptic weight through FORCE learning, while fully utilizing the complex dynamics of the POM/SWNT networks.

During learning, a temporal error between the output of the readout unit and supervisor signal $s(t)$,

$$e_{-}(t) = \mathbf{w}^{\mathrm{T}}(t-\Delta t)\mathbf{r}(t) - s(t),$$

was calculated ($\Delta t = 1$). The weight was then updated using

$$\mathbf{w}(t) = \mathbf{w}(t-\Delta t) - e_{-}(t)\mathbf{P}(t)\mathbf{r}(t),$$

where

$$\mathbf{P}(t) = \mathbf{P}(t-\Delta t) - \frac{\mathbf{P}(t-\Delta t)\mathbf{r}(t)\mathbf{r}^{\mathrm{T}}(t)\mathbf{P}(t-\Delta t)}{1+\mathbf{r}^{\mathrm{T}}(t)\mathbf{P}(t-\Delta t)\mathbf{r}(t)}.$$

The learning algorithm above is referred to as the FORCE learning algorithm[S2].

In Supplementary Fig. 2c, the NARMA10 sequence[S3] was used as the supervisor. The sequence was generated by

$$s(t+\Delta t) = 0.3s(t) + 0.05s(t)\left[\sum_{i=0}^{9} s(t-i\Delta t)\right] + 1.5u(t)u(t-9\Delta t) + 0.1,$$

where $u(t)$ consists of scalar random numbers with a uniform distribution in the intervals $[0,0.5]$. The reservoir system was trained, *i.e.* $\mathbf{w}$ of the linear readout unit was modified by the FORCE learning by comparing $z(t)$ and $s(t)$, during $t = 4{,}920{\sim}5{,}000$ steps, at which the array had already been charged, using the given parameter sets (the same parameter sets of the results shown in Fig. 4f). After learning, $\mathbf{w}$ was fixed, and the simulation was restarted using the same random seed used in the learning. Then, the reservoir output was observed with the fixed $\mathbf{w}$.

In Supplementary Figs. 2d and 2f, the NRMSD between the supervisor $s(t)$ and generated output $z(t)$ after learning, was calculated by using

$$\mathrm{NRMSD} = \frac{\mathrm{RMSD}}{\tilde{z}}, \mathrm{RMSD} \equiv \sqrt{\frac{1}{n}\sum_{i=0}^{n-1}\left(s(i\Delta t) - z(i\Delta t)\right)^2}, \tilde{z} \equiv \frac{1}{n}\sum_{i=0}^{n-1} z(i\Delta t),$$

where n represents the data length. In Supplementary Fig. 2d, the NRMSD versus R (50 and 100) and the signal length swept from 160 to 400 steps, where averaged NRMSD values over 50 trials for each R value with different random seeds are plotted.

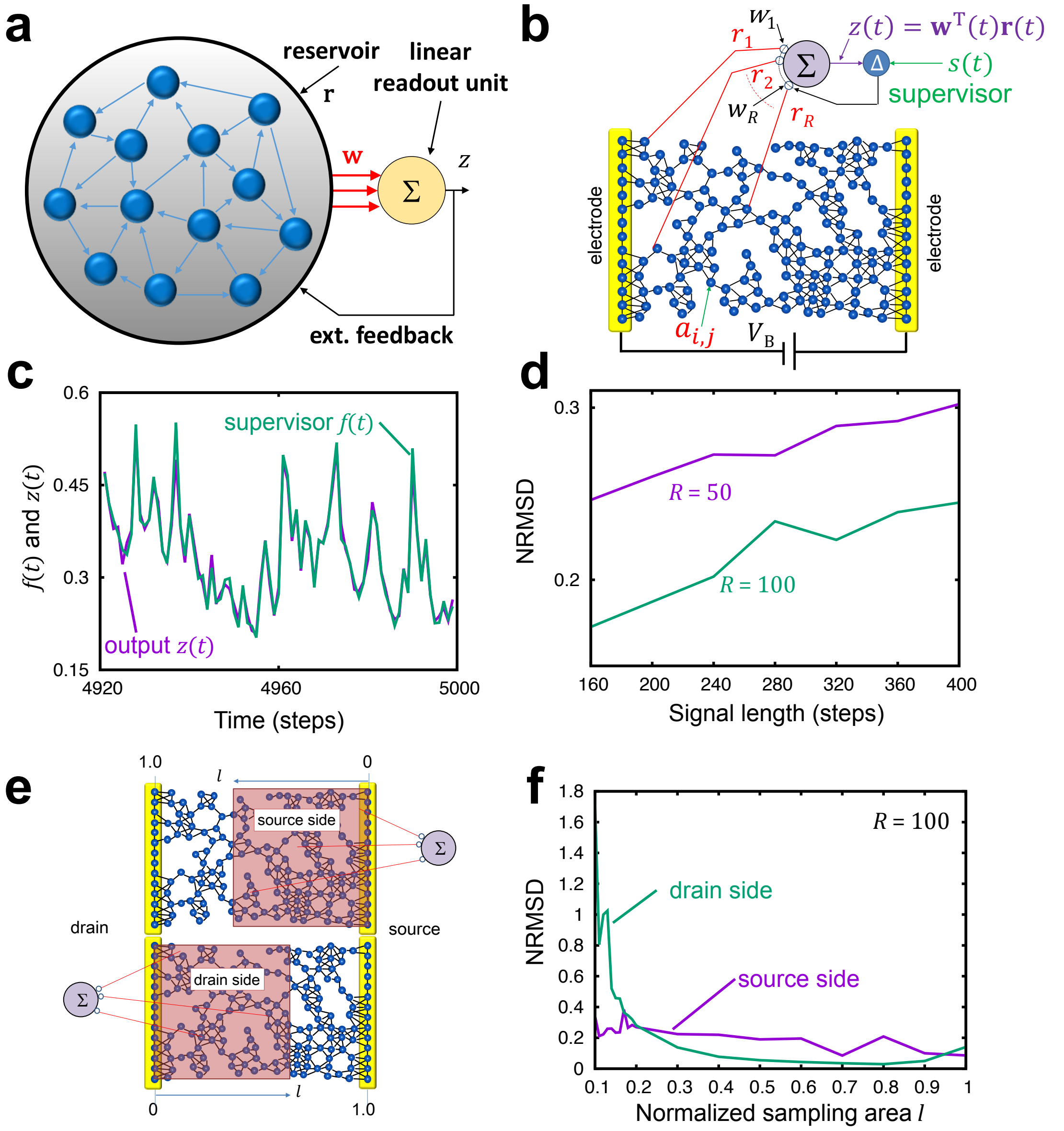

Supplementary Figure 2 | Demonstration of reservoir computing on the POM/SWNT network model. (**a**) Illustration of a standard reservoir system having external feedback, consisting of a recurrent generator network (blue spheres and directed arrows) with firing rates **r**, a linear readout unit Σ with output z through weights **w** (red), and the output z fed back to the reservoir. (**b**) Our reservoir system consisting of a 2D random network of POM particles (blue spheres) sandwiched between two electrodes (yellow bars). The charge of a POM particle positioned at (i, j) is represented by $a_{i,j}$. The weight **w** is modified by the output $z(t)$ and supervisor $s(t)$ during the learning phase, to attain $z(t) \approx s(t)$. (**c**) Time courses of NARMA10 benchmark sequence $s(t)$ (supervisor) used during the FORCE learning and the output of the readout unit $z(t)$ after learning. (**d**) NRMSD versus R (50: purple and 100: green) and the signal length from 160 to 400 steps. (**e**)

Two different readout-particle selection methods, where particles were randomly selected from areas beside the source electrode (upper, labelled by 'source side') or the drain electrode (bottom, 'drain side'). **(f)** Comparison of NRMSDs for source- and drain-side particle sampling as a function of the normalized sampling area (l).

Supplementary Notes 3: Possible experimental considerations for a reservoir computing system consisting of a POM/SWNT complex

To address the question of how a reservoir computing system can be made by using a real POM/SWNT complex network, we here provide two possible experimental methods: (1) attaching or depositing external electrodes onto a POM/SWNT network sheet and (2) fabricating a POM/SWNT network on a lithographically patterned multi-electrode array via electrophoresis.

In the investigations described in the main text, source and drain electrodes were used to supply power, to induce impulse generation. In Supplementary Figs. 2c, 2d, and 2f, 50 or 100 virtual readout probes ($R = 50$ and 100) were prepared; *i.e.* multi-channel non-perturbative probing was assumed. If one reads current through a probe, the probe itself acts as a drain terminal electrode, because the impedance of the probe must be extremely low to read small currents, and usually the probe is virtually grounded by a feedback amplifier. Thus, the power, signal input, and readout terminals have high degrees of freedom in the design of not only the POM/SWNT but also nano-electronic or molecular reservoirs.

(1) Attaching or depositing external electrodes onto a POM/SWNT network sheet: A top-contacted electrodes array is attached to a POM/SWNT network sheet, as shown in Supplementary Fig. 3. Although this system is easy to construct, the device size will be on the order of centimetres, and the number of readout (drain) terminals will be limited up to several hundred. It is easily conceivable fort an electrodes array to be stuck onto a POM/SWNT network sheet; however, the electrical contact will be insufficient.

(2) Fabricating a POM/SWNT network on a lithographically patterned multi-electrode array via electrophoresis method: This method is commonly used in neurophysiological experiments. Supplementary Figure 4 shows the basic schematics of a multi-electrode array consisting of metal shanks and lead lines embedded in an insulator, where only the top surfaces of the electrodes are exposed to the top surface of the insulator, as shown in Supplementary Figs. 4a and 4b. The electrode array can be sunk in a solution (Supplementary Fig. 4b). As mentioned in Supplementary Note 1, SWNTs can be aligned by being connected between the electrodes via electrophoresis.

By using the present CMOS processing technology, it is possible to fabricate an electrode array of submicron metal electrodes (*e.g.*, 0.3×0.3 um^2) with a 1-μm pitch, as shown in Supplementary Fig. 4c. Consequently, if an electrodes-array-embedded

POM/SWNT network, as shown in Supplementary Figs. 4b and 4c, were fabricated, they would be used as signal terminals; *i.e.* signal input (voltage supply) and signal output (current measurement) terminals. This technique has been employed in atomic switch networks, with the objective of constructing a reservoir computing system consisting of a silver atom switch[S5] .

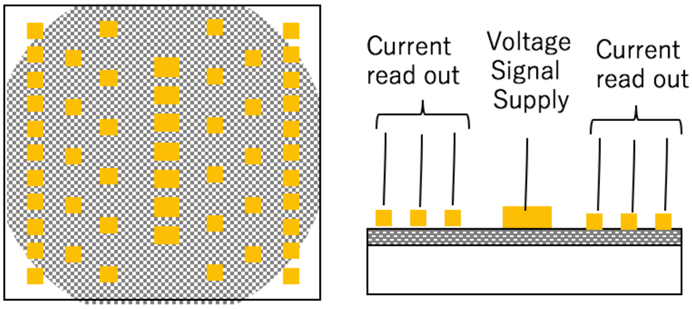

Supplementary Figure 3 | Reservoir device consisting of a POM/SWNT network sheet on an insulator substrate and top contacted electrodes.

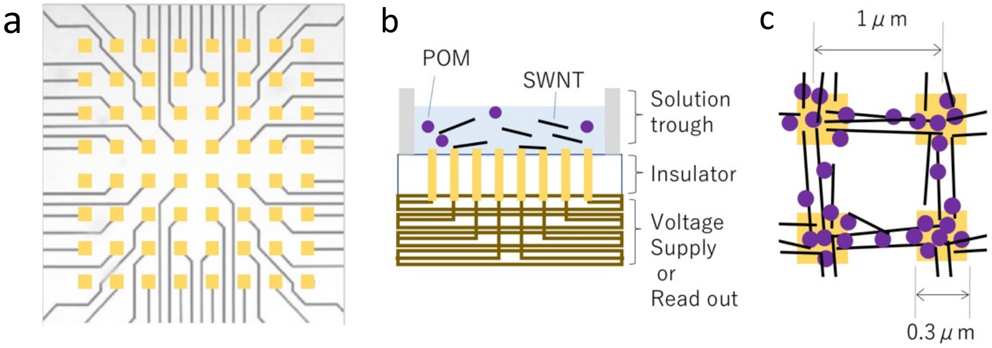

Supplementary Figure 4 | Reservoir device consisting of a lithographically patterned multi-electrode array and POM/SWNT complex. (**a**) Planer arrangement of the electrode array. (**b**) Cross-sectional view of the electrode array and the experimental settings for SWNT electrophoresis. (**c**) Enlarged schematic top view of POM/SWNT complex

Supplementary References

1 Fujii, H., Setiadi, A., Kuwahara, Y. & Akai-Kasaya, M. Single walled carbon nanotube-based stochastic resonance device with molecular self-noise source. *Appl. Phys. Lett.* **111**, 133501 (2017).

2 Sussillo, D. & Abbott, L. F. Generating Coherent Patterns of Activity from Chaotic Neural Networks. *Neuron* **63**, 544-557 (2009).

3 Appeltant, L. *et al.* Information processing using a single dynamical node as complex system. *Nat. Commun.* **2**, 468 (2011).

4 Blum, A. S. *et al.* Molecularly inherent voltage-controlled conductance switching. *Nat. Mater.* **4**, 167-172 (2005).

5 Demis, E. C. *et al.* Atomic switch networks-nanoarchitectonic design of a complex system for natural computing. *Nanotechnology* **26**, 204003 (2015).
